# Focusing on inflammation-driven pyroptosis in postherpetic neuralgia: from molecular mechanisms to therapeutic strategies

**DOI:** 10.3389/fimmu.2026.1883546

**Published:** 2026-07-01

**Authors:** Ying Zhang, Xinyao Li, Qianting Yin, Zihui Wang, Miao Tian, Aozhi Fang, Yuanyuan Li, Fan Wu, Jianqin Mao, Tengfei Qian, Shihua Li, Dongdong Qin

**Affiliations:** 1 The Fourth Affiliated Hospital, Yunnan University of Chinese Medicine, Yuxi, Yunnan, China; 2 College of Basic Medical Sciences, Yunnan University of Chinese Medicine, Kunming, Yunnan, China; 3 The Second Clinical Medical College, Yunnan University of Chinese Medicine, Kunming, Yunnan, China; 4 The First Clinical Medical College, Yunnan University of Chinese Medicine, Kunming, Yunnan, China; 5 The People’s Hospital of Mengzi, The Affiliated Hospital of Yunnan University of Chinese Medicine, Mengzi,Yunnan, China; 6 Yuxi People’s Hospital, The Sixth Affiliated Hospital of Kunming Medical University, Yuxi, Yunnan, China

**Keywords:** central sensitization, Inflammation, peripheral sensitization, postherpetic neuralgia, pyroptosis

## Abstract

Postherpetic neuralgia (PHN), the most frequent and recalcitrant sequela of herpes zoster, arises from a complex interplay between inflammatory cascades and programmed cell death. Emerging evidence indicates that pyroptosis—a form of inflammatory cell death mediated by the NLRP3/Caspase-1/GSDMD axis—drives both the onset and chronicity of PHN. This review outlines the mechanism of action of pyroptosis and its divergent effects in the peripheral *versus* the central nervous system. Varicella-zoster virus reactivation triggers initial inflammation, promoting NLRP3 inflammasome assembly and Caspase-1 activation. Activated Caspase-1 cleaves GSDMD, resulting in the formation of membrane pores and the release of pro-inflammatory mediators such as IL-1β and IL-18. Peripherally, pyroptosis of satellite glial cells sensitizes sensory neurons through paracrine signaling, leading to peripheral sensitization. Centrally, microglial and astrocytic pyroptosis amplifies neuroinflammation, with the resulting accumulation of reactive oxygen species inducing pyroptosis of GABAergic neurons. The loss of these inhibitory interneurons disrupts the excitatory-inhibitory balance, causing central sensitization. These interconnected processes establish a self-reinforcing inflammation–pyroptosis–pain cycle. Consequently, interventions targeting key pyroptosis nodes—including NLRP3, Caspase-1, GSDMD, and P2X7R—*via* small-molecule inhibitors, natural compounds, or non-pharmacological approaches such as electroacupuncture, hold significant therapeutic promise for PHN. This review systematically delineates the multifaceted mechanisms underlying the role of pyroptosis in this condition and highlights the therapeutic potential of blocking this pathway to counteract peripheral and central sensitization, thereby offering a novel framework and precision targets for PHN management.

## 1 Introduction

Postherpetic neuralgia (PHN) represents a form of chronic neuropathic pain (NP) that endures for at least 30 days following resolution of the herpes zoster rash, with its clinical features typically including burning, electric-shock-like, and stabbing pain (1). Epidemiological surveys indicate that the incidence of PHN is 21.10% in immunocompromised individuals and patients with autoimmune diseases, which is significantly higher than the 13.99% observed in the general population, with a particularly high prevalence in the elderly (2, 3). Beyond pain itself, PHN frequently coexists with sleep disruption, anxious and depressive symptoms, and other affective disturbances, which collectively impose a marked burden on patients’ daily functioning and well-being (4). From a therapeutic standpoint, the intricate and partly overlapping pathogenic processes that drive PHN have so far hindered the development of highly specific drug treatments (5).

The NOD-like receptor thermal protein domain associated protein 3 (NLRP3) inflammasome is a cytosolic multiprotein complex composed of the sensor NLRP3, the adapter protein apoptosis-associated speck-like protein containing a Caspase recruitment domain, and pro-Caspase-1 (6). The sensor NLRP3 itself contains three domains: the pyrin domain (PYD) responsible for recruiting apoptosis-associated speck-like protein containing a Caspase recruitment domain (ASC), the domain present in neuronal apoptosis inhibitor protein, MHC class II transcription activator, incompatibility locus protein from *Podospora anserina*, and telomerase-associated protein that mediates ATP-hydrolysis-dependent oligomerization, and the leucine-rich repeat (LRR) domain that maintains autoinhibition and senses danger signals (7). Upon activation, NLRP3 undergoes conformational changes and is transported via Golgi-derived vesicles to the microtubule-organizing center (MTOC), where it assembles into an active disk-like structure that subsequently recruits ASC and Caspase-1 (8).

The downstream effector protein gasdermin D (GSDMD) is the final executor of pyroptosis. Activated Caspase-1 cleaves GSDMD at a single site within its interdomain linker, releasing an N-terminal fragment with pore-forming activity (9). This fragment inserts into the plasma membrane, leading to cell swelling, membrane rupture, and the release of interleukin-1β (IL-1β) and interleukin-18 (IL-18) (10). Concomitantly, the released gasdermin D N-terminal fragment (N-GSDMD) oligomerizes and inserts into the plasma membrane to form transmembrane pores (9). Of note, an increase in NLRP3 protein level alone is insufficient to trigger GSDMD cleavage, and a second activation signal is required to promote NLRP3 oligomerization, ASC speck formation, and Caspase-1 activation (11).

Over recent years, the roles of inflammation and cell death in the pathogenesis of PHN have garnered increasing attention. Reactivation of latent varicella-zoster virus (VZV) within the dorsal root ganglia (DRG) triggers inflammation that activates signaling cascades, including the nuclear factor kappa-B (NF-κB) and mitogen-activated protein kinase (MAPK) (12–14) pathways. This activation subsequently initiates the assembly of the NLRP3 inflammasome which induces pyroptosis (15), an inflammatory form of programmed cell death. Pyroptosis significantly contributes to peripheral and central sensitization, thereby driving persistent pain (16, 17), which, in turn, can exacerbate inflammation and pyroptosis, ultimately establishing a vicious cycle “inflammation–pyroptosis–pain” cycle.

In this review, we systematically elucidate the mechanisms underlying the formation of this “inflammation–pyroptosis–pain” cycle, delineate the cellular and molecular mechanisms driving peripheral pain initiation and central pain amplification, and explore the therapeutic potential of targeting this network, thereby offering new insights into PHN management.

## 2 The inflammatory response in PHN

### 2.1 The peripheral inflammatory response

VZV is a neurotropic virus that typically remains latent within the DRG (18). Upon reactivation, it undergoes extensive replication and releases pathogen-associated molecular patterns (PAMPs), such as viral nucleic acids and envelope proteins, which are recognized by the host innate immune system. This recognition leads to immune infiltration of the site of viral latency by dendritic cells, macrophages, and other immune cells that express various Toll-like receptors (TLRs) (19). The binding of VZV nucleic acids to TLR9 causes its dimerization and activation, leading to the recruitment of myeloid differentiation primary response protein 88 (MyD88) and the subsequent activation of the NF-κB and MAPK signaling cascades. This results in the nuclear translocation of the transcription factors NF-κB and AP-1 and the consequent upregulation of the expression of NLRP3 and pro-inflammatory cytokines, including IL-1β, IL-6, and TNF-α (20, 21).

Concurrently, nerve injury resulting from VZV reactivation can directly activate satellite glial cells (SGCs), as can pro-inflammatory cytokines produced by immune cells. Once stimulated, SGCs. Activated SGCs, on one hand, amplify inflammation by releasing pro-inflammatory cytokines and chemokines (22). Additionally, extracellular ATP generated during the inflammatory response binds to the P2X7 purinergic receptor (P2X7R) (23). This binding induces conformational changes that open the ion channel, driving potassium efflux and sodium influx and calcium influxes, which serve as the triggering signal for NLRP3 inflammasome assembly (24).

### 2.2 The central inflammatory response

Central inflammation is characterized by the activation of microglia and astrocytes (25). During PHN, central inflammation is primarily initiated through two pathways. Peripheral nociceptive signals are transmitted along sensory neurons to the spinal dorsal horn, directly activating glial cells. Concurrently, viral activity alters permeability of the blood-brain barrier (BBB), allowing the entry of peripheral pro-inflammatory cytokines and damage-associated molecular patterns (DAMPs) to enter the central nervous system (CNS), where they resident glial cells (26). TNF-α and IL-1β can bind to TNFR and IL-1R on the microglial surface, respectively, triggering the MAPK and NF-κB pathways to upregulate the expression of pro-inflammatory cytokines and chemokines, thereby amplifying neuroinflammation (26, 27). Following VZV reactivation, microglia respond prior to astrocytes, releasing TNF-α, IL-1β, and CCL2. These molecules bind to TNFR, IL-1R, and CCR2 on the astrocyte surface, initiating signaling pathways including JAK-STAT3 and Notch-OLIG2. This cascade induces astrocyte hypertrophy and upregulates glial fibrillary acidic protein (GFAP) expression, prompting the transition from a resting state to a reactive phenotype (28, 29). The sustained inflammatory microenvironment induced by VZV drives astrocytes toward the neurotoxic A1 phenotype. These reactive astrocytes sustain neuroinflammation by secreting pro-inflammatory cytokines, chemokines, and neurotoxins, while directly sensitizing spinal dorsal horn neurons. This central sensitization occurs through the upregulation of Nav1.7 and NMDA receptors on the neuronal surface, which enhances synaptic transmission efficiency (30, 31). Furthermore, during neuroinflammation, peripheral ATP entering the spinal dorsal horn stimulates microglia and astrocytes to release endogenous ATP. Both glial cell types express P2X7R on their surface, and subsequent ATP binding to this receptor serves to amplify the central inflammatory response (32, 33).

### 2.3 Inflammation and pyroptosis

Upregulation of NLRP3 inflammasome assembly is observed in both the peripheral nervous system (PNS) and CNS during PHN, suggesting that pyroptosis occurs in both tissues (34). Pyroptosis, a GSDMD-dependent form of programmed cell death, pyroptosis is characterized by membrane pore formation, cellular swelling, cytoplasmic rupture, and the release of intracellular pro-inflammatory factors, including cytokines (IL-1β, IL-18, and TNF-α) and DAMPs such as ATP and HMGB1 (35). These cellular products activate neighboring cells, establishing a positive feedback loop that mutually amplifies both the inflammatory response and the pyroptotic cascade. Based on this close association between inflammation and pyroptosis, we next elaborate on the distinct mechanisms governing various types of pyroptosis within the peripheral and central nervous systems, alongside their specific roles in pain sensitization.

**3 Pyroptosis in the PNS and CNS**.

### 3.1 Pyroptosis in the PNS

#### 3.1.1 Pyroptosis of SGCs

SGCs are not only involved in maintaining ion homeostasis and providing metabolic support, while also playing a critical role in the development of NP (22). Direct evidence of SGC pyroptosis in PHN is currently lacking; however, the observed upregulation of the NLRP3 inflammasome within the PNS during PHN provides a molecular framework permissive for this process (36). In such a scenario, extracellular ATP within the VZV-reactivated DRG microenvironment would drive NLRP3/Caspase-1-dependent GSDMD cleavage, yielding the GSDMD N-terminal (N-GSDMD) fragment. This fragment would then insert into the plasma membrane to form pores, inducing membrane rupture and pyroptosis (37). Subsequent release of these cellular mediators—including IL-1β, IL-18, HMGB1, ATP and ATP—could then act on neighboring sensory neuron receptors *via* paracrine signaling. This interaction would enhance TRPV1 and P2X3 channel activity, reducing the action potential threshold, and inducing spontaneous firing, thereby leading to peripheral sensitization (38).

#### 3.1.2 Pyroptosis of sensory neurons

Whether sensory neurons can independently undergo functional pyroptosis remains a subject of debate. Mature sensory neurons, owing to their highly specialized structure and energy demands, may lack the full capacity to execute pyroptosis and may instead function more as responders to pyroptotic products rather than as initiators of pyroptosis (39). SGCs tightly wrap around primary sensory neuron cell bodies in the DRG, providing structural and functional support (40), and can act as a metabolic barrier regulating the neuronal microenvironment. Neurons surrounded by SGCs exhibit markedly reduced responsiveness to molecules such as ATP, glutamate, GABA, and bradykinin (41). During SGC pyroptosis, released mediators including IL-1β, IL-18, HMGB1, and ATP act directly on adjacent sensory neurons. Specifically, IL-1β binds to IL-1R, triggering p38 MAPK and Src kinase signaling, which increases the expression and function of TRPV1, Nav1.7, and P2X3 channels. This lowers the action potential threshold, inducing ectopic discharges and spontaneous pain (42, 43). Concurrently, ATP binds to P2X3 receptors to exacerbate neuronal depolarization, establishing a positive feedback loop that spreads abnormal excitability, while sensory neuron-derived ATP conversely acts on SGCs, worsening their pyroptosis (44, 45).

In summary, a self-reinforcing vicious cycle develops between SGCs and sensory neurons during pyroptosis. Pyroptotic products released by SGCs sensitize sensory neurons, while ATP released by sensory neurons further worsens SGC pyroptosis, and this mutual interaction leads to persistent peripheral sensitization.

### 3.2 Pyroptosis in the CNS

#### 3.2.1 Pyroptosis of microglia

In a spinal nerve injury model, microglia exhibited a marked increase in the levels of the pyroptosis marker Caspase-1, while those of the apoptosis marker Caspase-3 and the necroptosis marker p-RIPK3 remained unchanged, indicating that microglial pyroptosis was the primary driver of NP development (46). Although direct evidence of microglial pyroptosis in PHN is absent, VZV-induced neuroinflammation is known to enhances NLRP3 expression, while microglial Caspase-1 expression is elevated in spinal injury models, implying in PHN, microglia may undergo classical NLRP3/Caspase-1/GSDMD-mediated pyroptosis. In this cascade, Caspase-1 simultaneously cleaves GSDMD and processes pro-IL-1β and pro-IL-18 into their mature forms; the subsequent release of IL-1β and IL-18 further fuels neuroinflammation and lower nociceptive neuron thresholds, resulting in central sensitization (47).

#### 3.2.2 Pyroptosis of astrocytes

In a spinal cord injury model, astrocytes showed high expression of genes involved in classical NLRP3 inflammasome-driven pyroptosis, as identified by bioinformatics (48). These findings indicate that astrocytes can undergo NLRP3/Caspase-1/GSDMD-mediated pyroptosis, which is triggered by microglia-derived IL-1β, TNF-α, and HMGB1. Physiologically, astrocytes regulate synaptic glutamate and ion homeostasis, and their pyroptosis results in glutamate accumulation, overactivation of NMDA/AMPA receptors, calcium influx, and neuronal depolarization, which are essential for central sensitization (49). Notably, DAMPs released from pyroptotic astrocytes can also be recognized by TLRs or P2X7R on neighboring cells, which can induce another round of NLRP3 inflammasome assembly, thereby promoting further astrocytes toward pyroptosis. Through this cascade mechanism, localized nociceptive signaling within the spinal dorsal horn can propagate widely, ultimately rewiring pain transmission pathways on a global scale.

#### 3.2.3 Pyroptosis of GABAergic neurons

Dysfunction and selective loss of GABAergic neurons are core mechanisms underlying central sensitization and the maintenance of chronic pain (50). Hu et al. showed that mitofusin 2 (Mfn2) is crucial for mitochondrial integrity and energy balance, and causes mitochondrial fragmentation and excessive ROS production. Elevated ROS levels result in the activation of the NLRP3 inflammasome, which then triggers GABAergic neuron pyroptosis, thereby promoting chronic pain (37). ROS is a key DAMP released by pyroptotic astrocytes and microglia, ROS is a core component. In addition, factors such as IL-1β, ATP, and HMGB1 released from pyroptotic microglia can activate P2X7R on adjacent astrocytes, inducing the activation of endogenous NOX enzymes and the production of ROS, creating a positive feedback loop that further amplifies ROS levels (51).

These findings suggest that during PHN, ROS accumulation resulting from pyroptosis is a major upstream event in GABAergic neuron pyroptosis. The pyroptotic depletion of GABAergic interneurons—the primary inhibitory population in the spinal dorsal horn—disrupts central excitatory-inhibitory homeostasis, creating a disinhibited state that exacerbates central sensitization (52). Additionally, the material released from pyroptotic GABAergic neurons can also activate surrounding glial cells. An augmentation in GABAergic neuronal activity during acute stress precedes microglial activation, whereas chronic activation of these neurons can elicit microglial activation. Inhibition of this neuronal subtype ameliorates stress-induced microglial activation (53).This, in turn, impedes the resolution of inflammation within the spinal dorsal horn, ultimately contributing to the transition from acute to chronic pain.

### 3.3 The “inflammation–pyroptosis–pain” vicious cycle

**Figure 1:**
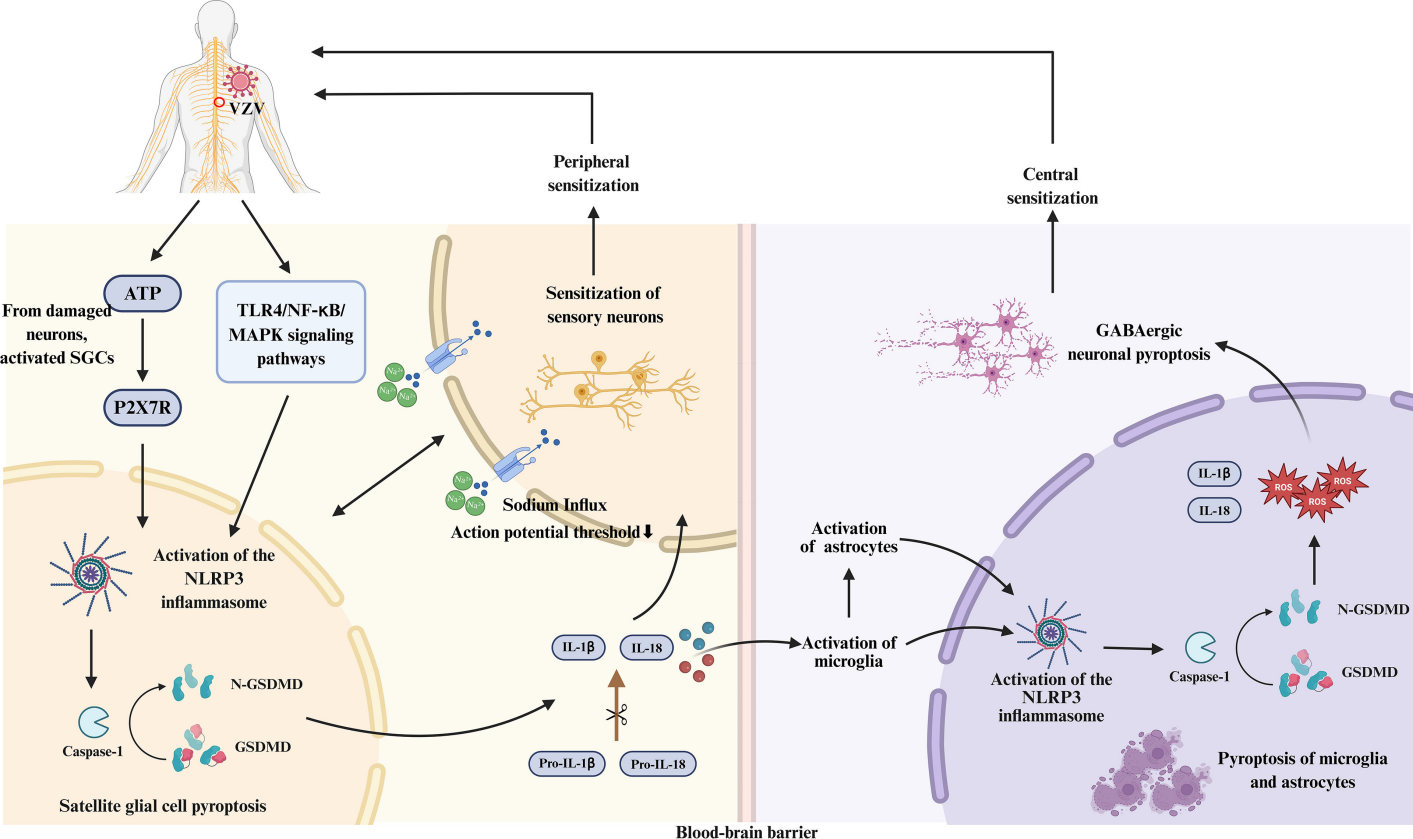
Schematic diagram of the molecular mechanisms underlying the “inflammation–pyroptosis–pain” vicious cycle in PHN. Upon VZV reactivation, the TLR4, NF-κB, and MAPK signaling pathways are activated, promoting NLRP3 inflammasome assembly and Caspase-1 activation. Caspase-1 then cleaves GSDMD, generating its N-terminal fragment (N-GSDMD), which forms membrane pores, and also processes pro-IL-1β and pro-IL-18 into mature IL-1β and IL-18. In the PNS, ATP derived from damaged neurons and activated SGCs acts on P2X7R on the cell membrane, providing an additional trigger for NLRP3 inflammasome activation. These events induce the pyroptosis of SGCs, accompanied by the release of IL-1β and IL-18. These mediators act on adjacent sensory neurons, increase sodium influx, and lower the action potential threshold, thereby driving peripheral sensitization. Peripheral nociceptive signals and pro-inflammatory mediators cross the compromised BBB and enter the CNS, where they activate microglia and astrocytes. When activated, these glial cells undergo NLRP3/Caspase-1/GSDMD-dependent pyroptosis. This self-reinforcing loop constitutes the “inflammation–pyroptosis–pain” vicious cycle, which underlies PHN chronification. VZV, varicella−zoster virus; TLR4, Toll-like receptor 4; NF-κB, nuclear factor kappa-B; MAPK, mitogen-activated protein kinase; NLRP3, NOD-like receptor family pyrin domain containing 3; GSDMD, gasdermin D; N-GSDMD, gasdermin D N-terminal fragment; IL-1β, interleukin-1 beta; IL-18 interleukin-18; PNS, periphery nervous system; CNS, central nervous system; ATP: adenosine triphosphate; P2X7R: P2X7 purinergic receptor; SGCs: satellite glial cells; BBB: blood–brain barrier.

Peripheral and central sensitization drive persistent pain and promote chronicity. HMGB1, a major DAMP, modulates pathological pain states, while pain signaling concurrently stimulates HMGB1 production and subsequent TLR4 activation (54). The resulting TLR4/NF-κB signaling maintains chronic pain through region-specific mechanisms. During PHN, this pathway upregulates TNF-α and IL-1β expression, thus accelerating pain development (55). Within the CNS, TLR4/NF-κB signaling activates microglia, sustaining chronic pain states (56). In NP, M1-polarized activated microglia release IL-1β and TNF-α and induce oxidative stress (57). These pro-inflammatory cytokines, alongside ROS, induce NLRP3 inflammasome assembly in microglia and astrocytes. This cascade initiates pyroptosis and intensifies neuroinflammation, establishing a self-sustaining “inflammation–pyroptosis–pain” cycle (**Figure 1**).

## 4 Therapeutic strategies targeting inflammation and pyroptosis

Inhibiting key molecular components within the “inflammation–pyroptosis–pain” vicious cycle described above offers a novel therapeutic strategy for PHN. Currently, research investigating pyroptosis specifically in PHN remains limited. **Table 1** summarizes the key mechanisms targeting inflammation and pyroptosis in relevant NP models, providing a theoretical framework for PHN.

**Table 1:** Therapeutic strategies targeting inflammation and pyroptosis aiming to alleviate pain in experimental models: effects and mechanisms.

Experimental model	Intervention drugs	Targets	Effects and mechanisms	References
Chronic constriction injury rat model	Bergapten	NLRP3 inflammasome, cleaved Caspase-1 and N-GSDMD	Inhibits NLRP3 inflammasome activation and downregulates mature Caspase-1, N-GSDMD, and pro-inflammatory cytokine levels, thereby alleviating pain	(58)
	Paeoniflorin	NLRP3 inflammasome	Alleviates pain by inhibiting NLRP3 inflammasome activation	(59)
	Ginkgolide B	NLRP3 inflammasome and Caspase-1	Inhibits NLRP3 inflammasome activation by inducing mitophagy, thereby alleviating pain	(60)
Chronic constrictive injury mouse model	Divanillyl sulfone	NLRP3 inflammasome	Inhibits NLRP3 inflammasome activation by inducing mitophagy, thereby alleviating pain	(61)
	Ginsenoside Rg1	NLRP3 inflammasome	Inhibits NLRP3 inflammasome activation by inducing mitophagy and also inhibits microglial pyroptosis, thus alleviating pain	(62)
Spared nerve injury mouse model	PD-1	NLRP3 inflammasome	Alleviates pain by inhibiting NLRP3 inflammasome activation and microglial pyroptosis to alleviate pain	(63)
T9 contusive spinal cord injury mouse model	VX-765	Caspase-1	Reduces neuroinflammation and interrupts the pyroptosis pathway by inhibiting Caspase-1 activation and IL-1β/IL-18 secretion, consequently alleviating pain	(64)
Spinal cord injury rat model and LPS/ATP-induced BV2 microglia cell model	Kaempferol	NLRP3 inflammasome, Caspase-1 and N-GSDMD	Downregulates the expression of the pyroptosis-related proteins NLRP3, Caspase-1, and N-GSDMD, and reduces IL-18 and IL-1β release, thereby alleviating neuroinflammation and treating pain	(65)
LPS and ATP-induced microglia pyroptosis *in vitro*	Dimethyl itaconate	NLRP3 inflammasome and GSDMD	Inhibits NLRP3 assembly and GSDMD cleavage, and suppresses NLRP3-dependent pyroptosis by inducing autophagy, thereby alleviating pain	(66)
PHN rat model	Brilliant blue G	P2X7R	Alleviates PHN by inhibiting P2X7R and reducing endoplasmic reticulum stress and pyroptosis	(16)
Rat neuropathic pain model	AK1780	P2X7R	Acts centrally, antagonizing ATP binding to P2X7R and inhibiting IL-1β release from microglia, producing analgesic effects	(67)
	Cilnidipine	P2X7R	Inhibits P2X7R-mediated calcium responses and IL-1β release, reversing mechanical hypersensitivity	(68)
TNF-α-stimulated astrocyte model and *in vivo* mouse spinal cord injury model	Indole-3-propionic acid	NF-κB/MAPK axis	Inhibits NF-κB/MAPK signaling by activating the aryl hydrocarbon receptor, reduces pro-inflammatory cytokine expression in astrocytes and alleviates pain	(69)
LPS -induced BV2 microglial model	ω-3 DPA	NF-κB/MAPK p38 pathway	Protects neurons from neuroinflammation-induced damage by balancing microglia M1/M2 polarization and inhibits the NF-κB and MAPK p38 pathways in microglia, thereby alleviating pain	(70)
	4-Methoxycinnamyl p-coumarate	NF-κB/MAPK	Alleviates pain by reducing ROS production in microglia and inhibiting NF-κB and MAPK activation	(71)
Vincristine-induced neuropathic pain	Celastrol	NF-κB	Inhibits astrocyte activation and reduces oxidative stress by blocking CaMKII phosphorylation, thereby alleviating pain	(72)
Spared nerve injury mouse model	Proanthocyanidins	ROS	Alleviates pain by scavenging ROS and relieving ROS-mediated inhibition of pyramidal cell excitability	(73)
Peripheral nerve injury-induced neuropathic pain model	Echinacoside and artesunate	Nrf2, Nedd4–2 and NaV1.7	Ameliorates oxidative stress by activating Nrf2, and specifically inhibits pathologically enhanced NaV1.7 current density, thereby alleviating pain	(74)
LPS-induced BV2 microglia ferroptosis *in vitro* and murine spinal cord injury model *in vivo*	Quercetin	ROS	Incorporated into hydrogels and injected into the injury site for sustained release, achieving ROS scavenging, reduction of neuroinflammation, and enhanced analgesic effects	(75)
Peripheral nerve-injury induced neuropathic pain model	Curcumin	ROS	Incorporated into hydrogels and injected into the injury site for sustained release, achieving ROS scavenging, reduction of neuroinflammation, and enhanced analgesic effects	(76)
Heterologous expression system and freshly isolated dorsal root ganglion neurons	Cannabinol	Nav1.7	Inhibits Nav1.7 activation, reduces Na^+^ influx, and decreases neuronal excitability, ultimately inhibiting peripheral sensitization and alleviating pain	(77)
SH-SY5Y neuroblastoma cell line and homology modeling/molecular dynamics	Tricyclic antidepressants	CaV2.2	Alleviates pain by blocking neuronal calcium channels and reducing neuronal excitability	(78)
*In vitro* assays and CaV2.2 homology model	N-Sulfonylphenoxazines	CaV2.2	Inhibits CaV2.2 with low micromolar activity, likely penetrates the blood−brain barrier, and shows enhanced plasma and liver microsome stability by binding within and above the channel’s selectivity filter, thereby hindering channel opening and alleviating pain	(79)
Neuropathic pain models	BoNTs	TRPV1	Relieve pain by reducing the expression of TRPV1 in sensory neurons, which is related to pain	(80)
Partial sciatic nerve ligation-induced neuropathic pain rat model	Tolperisone	Voltage-dependent sodium channels	Inhibits glutamate release from rat brain synaptosomes by blocking voltage-dependent sodium channels, thereby reducing central excitability and alleviating pain	(81)
Sural spared nerve injury mouse model	Hydrogel solution	GABA	Captures glutamate and converts it to GABA, reverses the inhibition of KCC2 expression in the spinal cord, ensures normal GABA receptor function, and can be used to repair the impaired GABAergic inhibitory system and alleviate pain	(82)
Chemotherapy-induced neuropathic pain models and diabetic neuropathic pain model in rodents	GABA uptake inhibitors	MGAT1 and mGAT4	Targets GABA transporters 1 and 4; the GABA uptake inhibitor compound 56a exhibits significant antinociceptive properties and shows potential for PHN treatment	(84)
Chronic constriction injury-induced neuropathic pain rat model	Repetitive transcranial magnetic stimulation	P2X7R and NLRP3 inflammasome	Disrupts integrin αvβ3-P2X7R interaction in the amygdala and inhibits NLRP3 inflammatory signaling pathway activation, thereby alleviating neuropathic pain	(85)
	Repetitive transcranial magnetic stimulation	Ifit3/Stat1 interaction	Downregulates the interaction between Ifit3 and Stat1, reduces neuroinflammation and microglial apoptosis, and alleviates neuropathic pain	(83)
	Electroacupuncture	NLRP3 inflammasome, Caspase-1 and N-GSDMD	Reduces neuroinflammation and improves pain by downregulating the levels expression of the NLRP3 inflammasome, mature Caspase-1, and N-GSDMD in microglia	(86)
	Spinal cord stimulation	Cav2.2	Produces significant analgesic effects by upregulating opioid receptor-1, thereby inhibiting Cav2.2 and suppressing the overexpression of its downstream neurotransmitters substance P and glutamate	(88)
Spinal nerve ligation-induced neuropathic pain rat model	Electroacupuncture	P2X7R	Alleviates pain by inhibiting P2X7R activation and reducing neuroinflammation	(87)

### 4.1 Targeting pyroptosis

#### 4.1.1 Targeting the NLRP3 inflammasome

The NLRP3 inflammasome amplifies neuroinflammation and initiates pyroptosis, rendering its inhibition a viable therapeutic strategy for PHN. In animal models of NP, paeoniflorin and bergapten alleviate NP by inhibiting NLRP3 inflammasome activation; bergapten additionally downregulates mature Caspase-1, GSDMD, and pro-inflammatory cytokine levels (58, 59). Divanillyl sulfone, ginkgolide B, and ginsenoside Rg1 suppress NLRP3 inflammasome activation by inducing mitophagy, and ginsenoside Rg1 further blocks microglial pyroptosis (60–62). Programmed cell death protein 1 (PD-1) similarly inhibits NLRP3 inflammasome activation and microglial pyroptosis (63). Although these strategies have been evaluated primarily in general NP models, they hold significant potential for PHN treatment, as PHN is a representative form of NP.

#### 4.1.2 Targeting the Caspase-1/GSDMD axis

The Caspase-1/GSDMD axis constitutes the core execution arm of the classical pyroptotic pathway, making it a direct target for blocking this form of cell death.In spinal cord injury models, VX-765 suppresses this axis by inhibiting Caspase-1 activation and IL-1β/IL-18 secretion, which reduces neuroinflammation and interrupts the pyroptotic cascade (64). Kaempferol downregulates the expression of NLRP3, Caspase-1, and N-GSDMD, thereby reducing IL-18 and IL-1β release, and alleviating neuroinflammation and NP (65). Dimethyl itaconate inhibits NLRP3 inflammasome assembly and GSDMD cleavage and suppresses NLRP3-dependent pyroptosis by inducing autophagy (66).

#### 4.1.3 Targeting P2X7R

When activated, the ATP-gated cation channel P2X7R initiates NLRP3 inflammasome assembly, thus bridging neuroinflammation and pyroptosis. In rat models of PHN, brilliant blue G (BBG) relieves pain by blocking P2X7R, attenuating endoplasmic reticulum (ER) stress, and decreasing pyroptosis (16). AK1780, a P2X7R antagonist with high CNS-penetrating potential, antagonizes the binding of ATP to P2X7R and inhibits IL-1β release, consequently producing analgesic effects (67). The calcium channel blocker cilnidipine inhibits P2X7R-mediated calcium responses and IL-1β release, which reverses mechanical hypersensitivity (68).

### 4.2 Targeting upstream regulatory pathways of pyroptosis

#### 4.2.1 Targeting the NF-κB/MAPK pathway

The NF-κB/MAPK pathway drives VZV-induced neuroinflammation, making it a viable target for indirectly blocking the onset of pyroptosis. In a spinal cord injury model, it was shown that indole-3-propionic acid activates the aryl hydrocarbon receptor, which inhibits this pathway and reduces pro-inflammatory cytokine production in astrocytes (69). Similarly, omega-3 docosapentaenoic acid (DPA) protects neurons from neuroinflammation-induced damage through the inhibition of the NF-κB and MAPK p38 pathways in microglia (70), while 4-methoxycinnamyl p-coumarate reduces ROS production and blocks both NF-κB and MAPK activation (71).

#### 4.2.2 Targeting oxidative stress

Oxidative stress both exacerbates neuroinflammation and acts upstream of NLRP3 inflammasome activation and, consequently, pyroptosis. Celastrol counters this cascade by blocking CaMKII phosphorylation, thereby suppressing astrocyte activation and oxidative stress (72). Proanthocyanidins alleviate pain by scavenging ROS and reversing the ROS-mediated inhibition of pyramidal cell excitability in the ventrolateral orbital cortex (73). Furthermore, the Nrf2-activating compounds echinacoside and artesunate ameliorate oxidative stress and specifically inhibit pathologically enhanced NaV1.7 current density (74). Injectable hydrogels are increasingly used for NP treatment. Antioxidants such as curcumin and quercetin can be incorporated into hydrogels for sustained release at the injury site, effectively scavenging ROS, reducing neuroinflammation, and enhancing analgesic effects (75, 76).

### 4.3 Coordinated interventions targeting peripheral–central sensitization

#### 4.3.1 Targeting pain-related ion channels

Pain-related ion channels play critical roles in regulating neuronal excitability and pain signal transmission after nerve injury. Cannabinol selectively inhibits sodium channel currents, which suppresses DRG neuron excitability (77). Tricyclic antidepressants reduce neuronal excitability *via* the blockade of neuronal calcium channels (78). N-sulfonylphenoxazines act as novel calcium channel blockers that impede the conformational changes required for channel opening (79). Botulinum neurotoxins decrease the expression of pain-associated molecules, such as TRPV1 and substance P, in sensory neurons (80). Tolperisone inhibits glutamate release through the blockade of voltage-dependent sodium channels, providing a mechanistic basis for its use in NP treatment (81).

#### 4.3.2 Restoring central excitatory–inhibitory balance

Pyroptosis of GABAergic neurons disrupts the central excitatory–inhibitory balance. Restoring this balance represents an effective therapeutic approach for PHN. A hydrogel composed of Pluronic F-127, recombinant GAD67, and the KCC2 enhancer CLP257 captures glutamate and converts it into GABA while reversing spinal KCC2 inhibition, an action that repairs the impaired GABAergic inhibitory system and alleviates NP (82). In the research literature on the mechanism of repetitive transcranial magnetic stimulation (rTMS) in treating neuropathic pain, through co-immunoprecipitation experiments, it has been proven that rTMS can downregulate the protein-protein interaction between Ifit3 and Stat1, reduce the binding and co-localization of the two proteins, thereby alleviating neuropathic pain (83). Similarly, vGABA uptake inhibitors exhibit significant antinociceptive properties in rodent NP models (84).

Beyond pharmacological strategies, physical therapy and non-pharmacological interventions offer distinct advantages for NP management and merit investigation in the context of PHN. Repetitive transcranial magnetic stimulation is a safe, non-invasive neuromodulation technique that eases pain by disrupting amygdala integrin αvβ3–P2X7R binding, which suppresses P2X7R-dependent NLRP3 inflammatory signaling (85). Electroacupuncture alleviates neuroinflammation and improves NP by inhibiting P2X7R activation or downregulating the levels of the NLRP3 inflammasome, mature Caspase-1, and N-GSDMD in microglia (86, 87). Finally, spinal cord stimulation produces significant analgesic effects by upregulating opioid receptor-1, which inhibits Cav2.2 and prevents the overexpression of its downstream neurotransmitters, namely, substance P and glutamate (88).

## 5 Conclusions and perspectives

PHN pathogenesis is driven by a self-perpetuating “inflammation–pyroptosis–pain” axis. VZV reactivation induces TLR4/NF-κB signaling, which upregulates the NLRP3 inflammasome and activates Caspase-1; this enzyme subsequently cleaves GSDMD, leading to the formation of membrane pores, the release of IL-1β, IL-18, and ATP, and the induction of pyroptosis in peripheral SGCs. This process sensitizes sensory neurons, resulting in peripheral sensitization. Within the CNS, microglial and astrocytic pyroptosis exacerbates neuroinflammation. Concurrently, ROS accumulation triggers the pyroptosis of GABAergic neurons, which disrupts the excitatory–inhibitory balance and establishes central sensitization. Intervening in this cascade offers therapeutic potential. The inhibition of NLRP3, Caspase-1, GSDMD, or P2X7R—alongside the application of natural compounds or non-pharmacological modalities—attenuates pyroptosis and reduces neuroinflammation.

The cell-specific mechanisms underlying the role of pyroptosis in PHN remain largely unelucidated, given that existing data derive primarily from general NP models. In addition to these molecular targeting methods, non-pharmacological neuroregulatory techniques, such as rTMS, may become a new type of non-invasive strategy for targeting NLRP3 inflammasome-GSDMD-mediated pyroptosis in PHN, thereby complementing drug intervention methods. However, the specific mechanism by which magnetic stimulation precisely alters protein binding, whether through direct conformational changes, mechanical transduction, or indirect regulation of protein expression, still needs to be further clarified.

Further investigation is necessary to define the spatiotemporal progression of pyroptosis across SGCs, microglia, astrocytes, and GABAergic neurons, as well as its specific interplay with apoptosis and autophagy. Systematic evaluation of the therapeutic window and safety profile of anti-pyroptosis strategies is also critical, as unchecked inhibition may elevate infection susceptibility or impede tissue repair. Future research should leverage single-cell sequencing, spatial transcriptomics, and multi-omics to dissect pyroptosis-associated networks. Finally, the development of cell-type-specific delivery systems and more clinically pertinent animal models of PHN is indispensable for translating these mechanistic insights into precision therapeutics.
